# Application of the Reverse Fragility Index to Statistically Nonsignificant Randomized Clinical Trial Results

**DOI:** 10.1001/jamanetworkopen.2020.12469

**Published:** 2020-08-05

**Authors:** Muhammad Shahzeb Khan, Gregg C. Fonarow, Tim Friede, Noman Lateef, Safi U. Khan, Stefan D. Anker, Frank E. Harrell, Javed Butler

**Affiliations:** 1 Department of Medicine, Cook County Health Sciences, Chicago, Illinois; 2 Division of Cardiology, Ronald Reagan–UCLA (University of California, Los Angeles) Medical Center, Los Angeles; 3 Department of Medical Statistics, University Medical Center Goettingen, Goettingen, Germany; 4 Department of Medicine, Creighton University, Omaha, Nebraska; 5 Department of Medicine, West Virginia University, Morgantown; 6 Department of Cardiology, and Berlin Institute of Health Center for Regenerative Therapies, German Centre for Cardiovascular Research partner site Berlin; Charité Universitätsmedizin Berlin, Berlin, Germany; 7 Department of Biostatistics, Vanderbilt University School of Medicine, Nashville, Tennessee; 8 Department of Medicine, University of Mississippi Medical Center, Jackson

## Abstract

**Importance:**

Interpreting randomized clinical trials (RCTs) and their clinical relevance is challenging when *P* values are either marginally above or below the *P* = .05 threshold.

**Objective:**

To use the concept of reverse fragility index (RFI) to provide a measure of confidence in the neutrality of RCT results when assessed from the clinical perspective.

**Design, Setting, and Participants:**

In this cross-sectional study, a MEDLINE search was conducted for RCTs published from January 1, 2013, to December 31, 2018, in *JAMA*, the *New England Journal of Medicine* (*NEJM*), and *The Lancet.* Eligible studies were phase 3 and 4 trials with 1:1 randomization and statistically nonsignificant binary primary end points. Data analysis was performed from August 1, 2019, to August 31, 2019.

**Exposures:**

Single vs multicenter enrollment, total number of events, private vs government funding, placebo vs active control, and time to event vs frequency data.

**Main Outcomes and Measures:**

The primary outcome was the median RFI with interquartile range (IQR) at the *P* = .05 threshold. Secondary outcomes were the number of RCTs in which the number of participants lost to follow-up was greater than the RFI; the median RFI with IQR at different *P* value thresholds; the median reverse fragility quotient with IQR; and the correlation between sample sizes, number of events, and *P* values of the RCT and RFI.

**Results:**

Of the 167 RCTs included, 76 (46%) were published in the *NEJM*, 50 (30%) in *JAMA,* and 41 (24%) in *The Lancet.* The median (IQR) sample size was 970 (470-3427) participants, and the median (IQR) number of events was 251 (105-570). The median (IQR) RFI at the *P* = .05 threshold was 8 (5-13). Fifty-seven RCTs (34%) had an RFI of 5 or lower, and in 68 RCTs (41%) the number of participants lost to follow-up was greater than the RFI. Trials with *P* values ranging from *P* = .06 to *P* = .10 had a median (IQR) RFI of 3 (2-4). When compared, median (IQR) RFIs were not statistically significant for single-center vs multicenter enrollment (5 [4-13] vs 8 [5-13]; *P* = .41), private vs government-funded studies (9 [5-13] vs 8 [5-13]; *P* = .34), and time-to-event primary end points vs frequency data (9 [5-14] vs 7 [4-13]; *P* = .43). The median (IQR) RFI at the *P* = .01 threshold was 12 (7-19) and at the *P* = .005 threshold was 14 (9-21).

**Conclusions and Relevance:**

This cross-sectional study found that a relatively small number of events (median of 8) had to change to move the primary end point of an RCT from nonsignificant to statistically significant. These findings emphasize the nuance required when interpreting trial results that did not meet prespecified significance thresholds.

## Key Points

**Question:**

In clinical trials with statistically nonsignificant primary end point results, what is the minimum number of events that must be changed to move the result from nonsignificant to statistically significant (ie, the reverse fragility index)?

**Findings:**

In this cross-sectional study of 167 randomized clinical trials with statistically nonsignificant results, the median reverse fragility index at a threshold of *P* = .05 was 8. A median of 8 events were required to change to enable a nonsignificant primary end point to become statistically significant.

**Meaning:**

Results of this cross-sectional study suggest that the reverse fragility index, along with effect sizes and associated 95% CIs, may provide a useful context for interpreting null clinical trial results.

## Teaser

This cross-sectional study describes application of the reverse fragility index in establishing the clinical relevance of published clinical trials with null results.

## Introduction

Interpreting randomized clinical trial (RCT) results and their clinical relevance when *P* values are marginally above or below the threshold of *P* = .05 is challenging.^1^ Although the clinical relevance may not be different, a *P* value marginally below the *P* = .05 threshold is usually accepted as a favorable finding in a trial, and a *P* value above the *P* = .05 threshold is considered an unfavorable result.^2^ Efficacy of an intervention should be evaluated comprehensively on the basis of the effect size measures, such as relative risk reduction or number needed to treat accompanied by *P* values and 95% CIs, but clinical research continues to emphasize the prespecified threshold of *P* = .05 when interpreting results. Such reliance on *P* values invites the risk of a type II error (ie, nonrejection of a false null hypothesis, which is also known as a false-negative or β error), especially in the presence of fewer events, small sample sizes, and/or limited follow-up times.^3,4,5^ Thus, it is critical to also evaluate the robustness of null trial results in cases in which the clinical consequences of a type II error are more important than those of a type I error (ie, rejection of a true null hypothesis, which is also known as a false-positive or α error), such as in disease states with high mortality and limited therapeutic options and with an acceptable intervention safety profile.

Robustness of statistically significant trials is often evaluated using the fragility index (FI), which is exclusively applied to trials that reach traditional statistical significance.^6,7,8,9,10,11,12,13,14,15^ Although a few studies have applied the FI to null clinical trial results of specific diseases,^7,15^ none of these previous studies have systematically assessed the robustness of a large number of statistically nonsignificant phase 3 to 4 trials with an emphasis on interpretability of null clinical trial results.

In this cross-sectional study, we used the concept of a reverse fragility index (RFI) to calculate the minimum number of events needed to change trial results from statistically nonsignificant to statistically significant. Our intent was to provide a measure of confidence in the neutrality of results when assessed from the clinical perspective.

## Methods

### Data Sources and Study Selection

In July 2019, 2 of us (N.L. and M.S.K) conducted a MEDLINE search for RCTs published in peer-reviewed general medical journals between January 1, 2013, and December 31, 2018. We used the following search specification: (“*Lancet* (London, England)”[Journal] OR “*The New England Journal of Medicine*”[Journal]) OR “*Journal of the American Medical Association*”[Journal]) AND (Randomized Controlled Trial [ptyp] AND (“2013/01/01”[PDAT]: “2018/12/31”[PDAT])). *JAMA*, the *New England Journal of Medicine*, and *The Lancet* were chosen because of their history of publishing landmark RCTs. No search restrictions were applied. Because this cross-sectional study used only publicly available data and did not involve patients, no institutional review board approval or informed consent was sought. We followed the Strengthening the Reporting of Observational Studies in Epidemiology (STROBE) reporting guideline.

Two of us (N.L. and M.S.K) screened all of the RCT titles and abstracts based on the predefined eligibility criteria, which included (1) phase 3 or 4 RCTs, (2) 2-arm studies with 1:1 randomization, and (3) statistically nonsignificant binary primary end points. Letters, editorials, systematic reviews or meta-analyses, opinions, observational studies, economic or cost-effective analyses of RCTs, cohort nonrandomized studies, quasi-randomized trials, and post hoc or secondary analyses of previously reported RCTs were excluded. Only RCTs were considered because the concept of fragility is not applicable to non-RCTs owing to the presence of confounders and selection bias.^8^ Moreover, only RCTs that reported dichotomous outcomes were considered because an RFI, defined as the number of converted cases needed to make a nonsignificant result significant, cannot be applied for continuous variables.^8^

### Data Extraction and Outcomes

We used a prespecified data collection form to extract data from all RCTs. In cases of discrepancies, another independent reviewer (S.U.K) reviewed the data and adjudicated. Data abstracted included the study outcome, event rates, sample sizes of comparative groups, location of the trial, type of blinding, single-center vs multicenter enrollment, type of funding (government or private), number of participants lost to follow-up, follow-up duration, and acknowledgment of the potential for underpowering in the discussion or conclusion section. For time-to-event outcomes, the total number of events in each group over the entire follow-up period was included. For the number of participants lost to follow-up, only those who were truly lost to follow-up were considered, and other diminution factors of the denominator, such as deaths, were not accounted for because they were considered outcome events. In this study, the primary outcome was the median (interquartile range [IQR]) RFI at the *P* = .05 threshold. The secondary outcomes were the number of RCTs in which the number of participants lost to follow-up was greater than the RFI; the median RFI with IQR at different *P* value thresholds; the median reverse fragility quotient (RFQ) with IQR; and the correlation between sample sizes, number of events, and *P* values of the RCT and RFI.

### Statistical Analysis

The RFI was calculated by subtracting events from the group with a lower number of events while simultaneously adding nonevents to the same group to keep the number of participants constant until the Fisher exact test 2-sided *P* value became less than .05. Because of the recent proposals to lower the *P* value threshold,^16,17^ we also calculated the RFI at the *P* = .01 and *P* = .005 thresholds. These calculations were carried out in the same method as already described except that events were subtracted from the group with a lower number of events while simultaneously adding nonevents to the same group to keep the number of participants constant until the Fisher exact test 2-sided *P* value became less than .01 for the *P* = .01 threshold and .005 for the *P* = .005 threshold. This calculation allowed us to see how the magnitude of the RFI changed if the *P* value threshold was made more rigorous. We calculated the RFI only for primary end points because RCTs are powered to detect the treatment effect for the primary end point. Thus, the relevance of fragility might be limited for secondary outcomes.^8^

A lower RFI indicates less statistical robustness and vulnerability to move from statistical nonsignificance to significance on the basis of a few events. Currently, no cutoff is deemed acceptable for fragility. The number of participants lost to follow-up was compared with the RFI for each trial given that loss to follow-up was associated with both the number of study participants at risk and the number of recorded events. Trials in which the number of participants lost to follow-up is greater than the RFI is concerning because factoring in the unknown outcomes of participants who are lost to follow-up can easily alter the significance of the results.^18,19^ Moreover, we calculated the RFQ, which is the RFI divided by the sample size, because the RFI is an absolute measure and does not account for the sample size, making it difficult to compare the reverse fragility of different RCTs or to set a standard RFI value.^20^ Knowing the RFQ enables the assessment of the proportion of events that must be changed to move the results from nonsignificant to significant. For example, trial X has an RFI of 2 and a sample size of 100, whereas trial Y has an RFI of 2 and a sample size of 200. Although both trials have the same RFI, we can use the RFQ to gauge which trial is relatively more fragile. Trial X has an RFQ of 0.02, which means that approximately 2 events per 100 participants are needed to change the significance of the results. Trial Y has an RFQ of 0.01, which means that the nonsignificance of trial Y is contingent on approximately 1 event per 100 participants, suggesting that trial Y is relatively more fragile. A smaller RFQ indicates a less robust study. In addition, we calculated the proportion of RCTs with an RFI that was 1% or less of the total sample size.

We reported the overall RFIs as medians with IQRs. Spearman rank correlation coefficient was used to assess the correlation between sample size, number of events, and *P* value of the RCT and RFI. The Kruskal-Wallis statistic was applied to detect the association between RFIs and nominal variables in more than 2 groups, and the Mann-Whitney rank sum test was used for the association in 2 groups (sensitivity analysis). A 2-tailed *P* < .05 indicated statistical significance for all assessments. All analyses were performed with R, version 3.51 (R Foundation for Statistical Computing) and Excel, version 14.1.3 (Microsoft Corp). Data analysis was conducted from August 1, 2019, to August 31, 2019.

## Results

**Figure 1.**
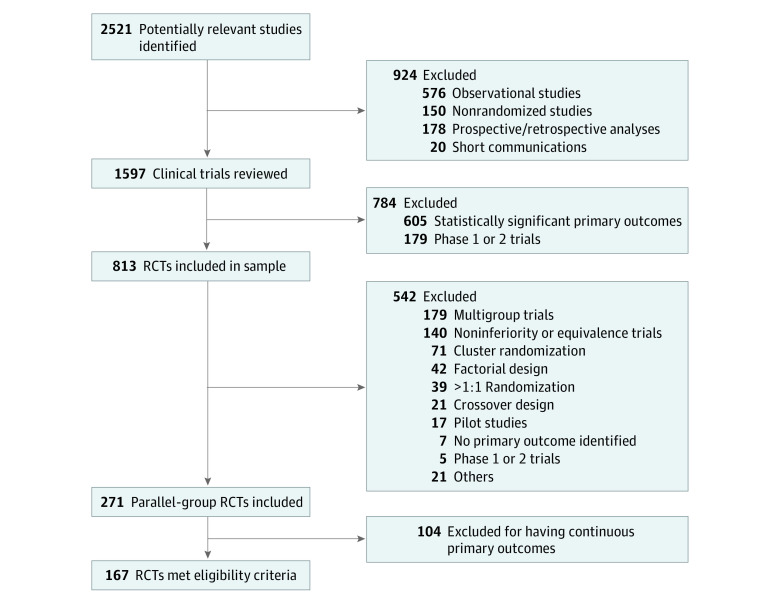
Flowchart of Search Strategy RCT indicates randomized clinical trial.

Of the 2521 potentially relevant studies identified, 167 RCTs (7%) met the eligibility criteria (eTable in the Supplement); Figure 1 shows the literature search strategy. The characteristics of the included RCTs are presented in Table 1. All trials used *P* ≤ .05 as the threshold of significance, and the range of sample sizes was from 48 to 31 999 participants. Of the 167 RCTs, 76 (46%) were published in the *New England Journal of Medicine*, 50 (30%) in *JAMA*, and 41 (24%) in *The Lancet*. The median (IQR) follow-up time was 6 (2-20) months, whereas the median (IQR) number of participants lost to follow-up was 38 (19-79). The median (IQR) total sample size was 970 (470-3427) patients, with 472 (226-1717) participants in the control groups and 459 (228-1707) participants in the intervention groups. The median (IQR) total number of events was 251 (105-570), with 127 (54-286) events in the control groups and 128 (50-279) events in the intervention groups. Eighteen RCTs (11%) had *P* values between *P* = .06 and *P* = .10. Mortality was assessed as a primary end point in 90 RCTs (54%). Sixty-four RCTs (38%) had time-to-event primary end points, and 49 trials (29%) acknowledged the potential for being underpowered in their discussion or conclusion section.

**Table 1.** Characteristics of the Included Trials

RCT characteristic	No. (%)^a^
Total No.	167
Sample size, median (IQR)	
Total	970 (470-3427)
Intervention	459 (228-1707)
Control	472 (226-1717)
Follow-up, median (IQR), mo	6 (2-20)
No. of participants lost to follow-up, median (IQR)	38 (19-79)
Events, median (IQR)	
Total	251 (105-570)
Intervention	128 (50-279)
Control	127 (54-286)
Mortality as primary end point	
Total	90 (54)
Single	30 (18)
Component of composite end point	60 (36)
Journal	
* NEJM*	76 (46)
* JAMA*	50 (30)
* The Lancet*	41 (24)
Country of origin	
Multiple countries	54 (32)
US	21 (13)
Europe	62 (37)
Asia	10 (6)
Others	20 (12)
Blinding type	
Single-blinded	15 (9)
Double-blinded	85 (51)
Unblinded	63 (38)
Not reported	4 (2)
Enrollment type	
Single center	9 (5)
Multicenter	158 (95)
No. of centers, median (IQR)	29 (15-73)
Intervention	
Pharmaceutical	92 (55)
Surgical	7 (4)
Other	68 (41)
Control group	
Placebo	66 (40)
Active	101 (60)
Funding type	
Government	84 (50)
Private	46 (28)
Both	37 (22)
Cardiovascular specialty	
Yes	68 (41)
No	99 (59)
Time-to-event end point	
Yes	64 (38)
No	103 (62)
Time to event, median (IQR), mo	28 (8-60)

Abbreviations: IQR, interquartile range; *NEJM*, *New England Journal of Medicine*; RCT, randomized clinical trial.

^a^ Values are given as number (percentage) unless otherwise stated.

### Reverse Fragility Index at *P* = .05 Threshold 

The median (IQR) RFI of the 167 trials was 8 (5-13), indicating that a median of 8 events was required to change the results of the primary end point from nonsignificant to significant. Fifty-seven RCTs (34%) had an RFI of 5 or lower. In 68 RCTs (41%), the number of participants lost to follow-up was greater than the RFI (Table 2). In such trials, the median (IQR) RFI was 8.5 (5-13), and the median (IQR) number of participants lost to follow-up was 37.5 (18-78). When compared, median (IQR) RFIs were not statistically significant for single center vs multicenter enrollment (5 [4-13] vs 8 [5-13]; *P* = .41), private vs government funding (9 [5-13] vs 8 [5-13]; *P* = .34), and time-to-event primary end points vs frequency data (9 [5-14] vs 7 [4-13]; *P* = .43) (Table 3).

**Table 2.** Comparison of RFI With Number of Participants Lost to Follow-up for Each RFI Range

RFI range	No. (%)	
	RCTs	LFU > RFI
1-5	57	22 (38)
6-10	44	18 (41)
11-15	36	14 (39)
16-20	15	6 (40)
21-25	7	4 (57)
26-30	5	2 (40)
31-35	2	1 (50)
36-40	1	1 (100)

Abbreviations: LFU, lost to follow-up; RCTs, randomized clinical trials; RFI, reverse fragility index.

**Table 3.** Number of Trials in Each Category With Median RFI and RFQ

Category	No. of trials	Median RFI (IQR)	*P* value	Median RFQ (IQR)	*P* value
Journal					
* NEJM*	76	11 (6-16)	.01	0.007 (0.002-0.011)	.02
* JAMA*	50	5 (4-9.25)		0.012 (0.005-0.022)	
* The Lancet*	41	8 (4-12.50)		0.008 (0.003-0.015)	
Country of origin					
Multiple countries	54	12 (6.75-17.25)	.06	0.003 (0.002-0.008)	<.001
US	21	8 (4.50-9.50)		0.013 (0.006-0.020)	
Europe	62	7 (4.75-13)		0.012 (0.007-0.018)	
Asia	10	13 (4.75-16.25)		0.009 (0.007-0.014)	
Others	20	7.50 (4-10.75)		0.004 (0.001-0.011)	
Blinding type					
Single-blinded	15	9 (4-12)	.94	0.007 (0.002-0.013)	.29
Double-blinded	85	8 (4-13)		0.006 (0.002-0.016)	
Unblinded	63	9 (5-13)		0.010 (0.004-0.017)	
Not reported	4	7 (5.25-12.50)		0.007 (0.004-0.024)	
Enrollment type					
Single center	9	5 (4-13)	.41	0.006 (0.003-0.011)	.47
Multicenter	158	8 (5-13)		0.008 (0.003-0.016)	
Intervention					
Pharmaceutical	92	8 (4-13)	.57	0.006 (0.002-0.015)	.50
Surgical	7	5 (3-8)		0.007 (0.004-0.027)	
Other	68	11 (5-14)		0.009 (0.005-0.016)	
Control group					
Placebo	66	8 (4-13)	.70	0.008 (0.002-0.016)	.48
Active	101	9 (5-13)		0.008 (0.003-0.015)	
Funding type					
Government	84	8 (5-13)	.34	0.009 (0.004-0.015)	<.001
Private	46	9 (5-13)		0.003 (0.001-0.008)	
Both	37	7 (4-12.50)		0.013 (0.002-0.023)	
Cardiovascular trial					
Yes	68	11 (6-17)	.01	0.003 (0.002-0.009)	<.001
No	99	7 (4-12)		0.011 (0.005-0.019)	
Time to event analysis					
Yes	64	9 (5.25-13.75)	.43	0.003 (0.002-0.011)	<.001
No	103	7 (4-13)		0.010 (0.005-0.018)	
Range of RFI					
1-5	57	4 (3-5)	<.001	0.006 (0.002-0.018)	.03
6-10	44	8 (7-9)		0.011 (0.004-0.018)	
11-15	36	13 (12-13)		0.013 (0.005-0.017)	
16-20	15	17 (16-19)		0.005 (0.003-0.010)	
21-25	7	23 (22-23)		0.004 (0.002-0.006)	
26-30	5	27 (26.50-40)		0.003 (0.002-0.005)	
31-35	2	34 (34-34)		0.002 (0.002-0.003)	
36-40	1	37 (37-37)		0.003 (0.003-0.003)	
*P* value categories					
.06-.10	18	3 (2-4)	<.001	0.001 (0.000-0.002)	<.001
.11-.20	19	4 (3-8)		0.005 (0.000-0.008)	
.21-.30	11	8 (4-16)		0.008 (0.002-0.010)	
.31-.40	11	5 (4-9)		0.006 (0.004-0.014)	
.41-.50	21	7.50 (5-10.75)		0.011 (0.005-0.021)	
>.50	87	12 (8-15)		0.012 (0.005-0.018)	

Abbreviations: IQR, interquartile range; *NEJM*, *New England Journal of Medicine*; RFI, reverse fragility index; RFQ, reverse fragility quotient.

**Figure 2.**
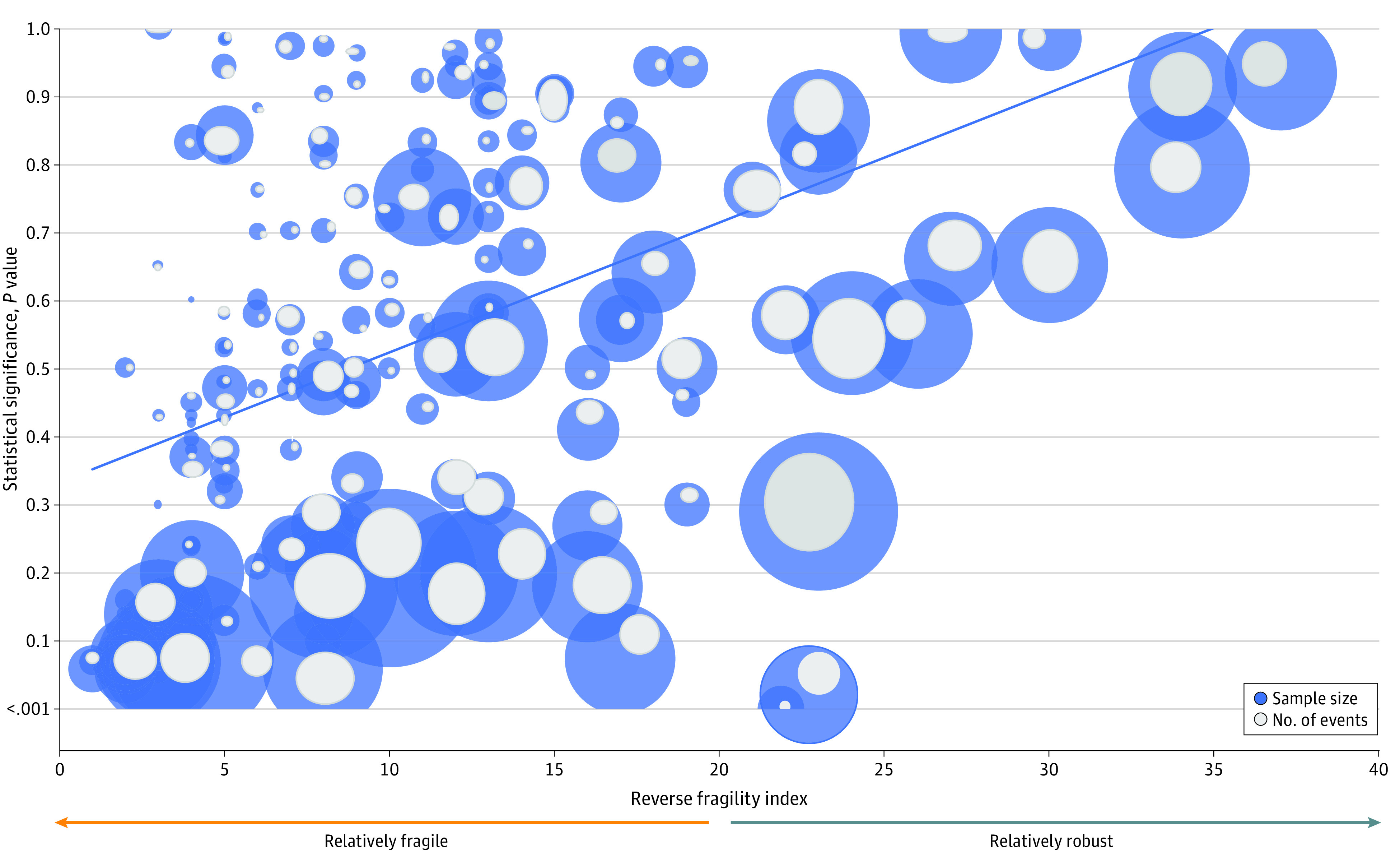
Scatterplot of the Correlation Between Reverse Fragility Index and *P* Value, Sample Size, and Total Number of Events The size of the blue circles is proportional to the sample sizes. The size of the gray shading inside the blue circles is proportional to the number of events.

Trials with *P* values ranging from *P* = .06 to *P* = .10 had a median (IQR) RFI of 3 (2-4). The RFIs and *P* values were statistically significantly correlated (Spearman correlation coefficient [*r*] = 0.54; 95% CI, 0.42-0.64; *P* < .001). Similarly, the RFI and total number of events demonstrated a statistically significant correlation (*r* = 0.65; 95% CI, 0.55-0.73; *P* < .001). Figure 2 shows the scatterplots of the correlation between RFI and *P* value, sample size, and total number of events.

### Reverse Fragility Quotient at *P* = .05 Threshold

The median (IQR) RFQ was 0.008 (0.0025-0.0155), indicating that the nonsignificance of the results was contingent on only 0.8 events per 100 participants. The median (IQR) RFQ of trials with an RFI lower than the number of participants lost to follow-up was 0.004 (0.0018-0.0118), whereas the median (IQR) RFQ of the remaining trials was 0.01 (0.004-0.0180). Of the 167 RCTs, 124 (74%) had an RFI that was 1% or less of the total sample size, and 107 RCTs (64%) had an RFI that was 2% or less of the total number of events.

### Reverse Fragility Index at Different *P* Value Thresholds

The median (IQR) RFI at the *P* = .01 threshold was 12 (7-19) and at the *P* = .005 threshold was 14 (9-21). Only 11 RCTs at the *P* = .01 threshold and 7 RCTs at the *P* = .005 threshold had an RFI of 5 or lower. eFigures 1 and 2 in the Supplement show the number of RCTs within each range of RFI at different *P* value thresholds. The RFI at both thresholds had a statistically significant correlation with sample size (*r* = 0.83, *P* < .001 at the *P* = .01 threshold; *r* = 0.69, *P* < .001 at the *P* = .005 threshold) and total number of events (*r* = 0.83, *P* < .001 at the *P* = .01 threshold; *r* = 0.86, *P* < .001 at the *P* = .005 threshold) (eFigures 3 to 6 in the Supplement).

### Reverse Fragility Quotient at *P* = .01 and *P* = .005 Thresholds 

At the *P* = .01 threshold, the median (IQR) RFQ was 0.0113 (0.0042-0.0217), suggesting that the nonsignificance of the results was contingent on approximately 1 event per 100 participants. The median (IQR) RFQ at the *P* = .005 threshold was 0.6937 (0.5960-0.7472), indicating that nonsignificance of the results was contingent on approximately 70 events per 100 participants. Of the 167 RCTs, 98 RCTs (59%) had an RFI that was 1% or less of the total sample size at the *P* = .01 threshold compared with no trials for the *P* = .005 threshold.

## Discussion

This study found that approximately one-third of null RCTs published in journals with a high impact factor had an RFI of 5 or lower and that, in 41% of the trials, the number of participants lost to follow-up was greater than the RFI. The RFI was particularly low in trials with *P* values that ranged from *P* = .06 to *P* = .10, and a strong correlation between *P* values and RFIs was noted. The median RFQ at the *P* = .05 threshold was 0.008, indicating that nonsignificance of the results was only contingent on 0.8 events per 100 participants.

The FI has been reported for many specialties, subspecialties, and clinical care guidelines.^6,7,8,9,10,11,12,13,14,15,21^ The median (IQR) FI score reported was 26 (0-118) in heart failure trials,^7^ 16 (4-29) in diabetes treatment guidelines,^15^ and 5 (1-9) in antithrombotic therapy^21^ for venous thromboembolism guidelines. Similarly, a review of almost 400 medical and surgical trials published in the *New England Journal of Medicine*, *JAMA*, and *The Lancet* showed that the median (IQR) FI score was 8 (0-109) and that, in 53% of these trials, the number of participants lost to follow-up was greater than the FI score.^6^ These results are similar to our RFI findings.

Statistical nonsignificance interpreted in the form of *P* values can misinform and may be disconnected from the real effect of the intervention.^22^ Clinicians who lack in-depth knowledge of statistical and trial design parameters, such as event rates, power to detect estimate differences, number of participants lost to follow-up, and follow-up duration to allow a sufficient time window for outcome differences to emerge, may have a tendency to base their conclusions solely on *P* values. This tendency may be relevant when interpreting the results of interventional or surgical trials in which a small initial excess of events in the intervention group could be switched if the intervention is advantageous. For example, the STICH (Surgical Treatment for Ischemic Heart Failure) trial^23^ was statistically nonsignificant at 5 years of follow-up with an RFI of 5, but the results became statistically significant in the 10-year extended follow-up trial.^24^ Another example is the EOLIA (Extracorporeal Membrane Oxygenation to Rescue Acute Lung Injury in Severe Acute Respiratory Distress Syndrome) trial.^25^ In the EOLIA study, although the primary outcome of 60-day mortality was not statistically significantly different between veno-venous extracorporeal membrane oxygenation and conventional therapy (35% vs 46%; relative risk, 0.76; 95% CI, 0.55-1.04; *P* = .09), the RFI was lower than 5 and clear advantages were seen in most secondary outcomes. In the present context, the RFI provides readers, participants, and clinicians alike with an alternative way to understand null trial results and to convey uncertainty numerically to guide clinical interpretation and future research.

We propose carefully assessing trials with *P* values ranging from *P* = .05 to *P* = .10 to avoid potentially overlooking advantageous interventions given that a type II error might be more damaging than a type I error. No specific cutoff value of FI or RFI exists that can be used to define acceptable robustness. A high RFI value does not necessarily indicate a robust result, and a low RFI may not mean the result is not robust. For instance, if the number of participants lost to follow-up is greater than the RFI, the RCT results should still be viewed with skepticism even if the RFI value is high. Therefore, considering different biases when interpreting trial results is important. Moreover, because RFIs are strongly correlated with *P* values and sample sizes, RFIs may not be used in assessing the robustness of trials without a broader context. Nevertheless, RFI is an intuitive index for interpreting null trial results in addition to effect sizes and 95% CIs.

Recently, some researchers have proposed to change the statistical significance threshold from *P* = .05 to *P* = .005 to guard against false-positive results.^16,17^ Thus, we calculated the RFIs at different *P* value thresholds to allow stakeholders to gauge how some of these proposals will play out using the concept of RFI. We found that the median RFI increased from 8 to 12 at the threshold of *P* = .01 and to 14 at the threshold of *P* = .005. Changing the statistical significance threshold does not solve the primary problem of *P* values being viewed in a dichotomized manner. Moreover, using a rigorous threshold will make it challenging for investigators and sponsors to come up with breakthrough therapeutics and may make it easier to overlook many potential treatments. In line with the recommendation of the American Statistical Association, we suggest the continued interpretation of *P* values rather than a distinct demarcation based on the prespecified value of *P* = .05, especially when borderline results are obtained at trials.^26^ Furthermore, all *P* values should be accompanied by point estimates and margins of error as recommended by the statistical guidelines of *JAMA* and the *New England Journal of Medicine*.^27,28^ Despite what *P* value threshold is taken, RFI may serve as an additional metric to show how statistical significance can be missed.

The RFI does not solve the more complex statistical issues underlying clinical practice but is basically an extension of a frequentist approach to trial analysis. It also has the many inherent limitations of null hypothesis testing. Null hypothesis statistical testing assumes a single hypothesis and generally brings about the use of a dichotomized approach of rejecting or not rejecting the null hypothesis on the basis of a single study.^29,30,31^ Thus, RFI and FI have been criticized for perpetuating the dichotomization of *P* values and have been described as just a restatement of a *P* value.^18^ Despite these claims, RFI is important because it provides an easy, quick way to see the gray in a typically black-or-white interpretation of results; it also helps illustrate the drawback of the use of *P* value thresholds in defining the statistical significance of treatment effects by showing their relative fragility. Compared with null hypothesis statistical testing, a bayesian approach offers a natural framework for incorporating additional information, including data from other trials or elicited expert opinion, into the analyses. Formally, this outcome is achieved by so-called priori distributions that reflect the additional information. In this way, bayesian approaches can facilitate the interpretation of trials.^32^ However, although bayesian approaches make sense theoretically, their application is sometimes hindered by the need to prespecify prior distributions. Moreover, although a bayesian approach is attractive, most clinical research is currently based on frequentist approaches; in such a case, RFI is a useful supplemental method. Therefore, large RCTs with a high number of events should be advocated for generating robust evidence.

### Limitations

This study has several limitations. First, use of the RFI concept was limited to null trial results, which had 1:1 randomization and primary dichotomous end points. Clinically important continuous end points were excluded from the study. Second, the use of Fisher exact test in calculating the RFI was crude because some of the included RCTs analyzed data in models with covariates or time-to-event techniques in which the original numbers, if analyzed with Fisher exact test, would not have the same *P* value as the published study. Thus, the RFIs did not account for the contribution of time to the difference in treatment effects. However, Walsh et al^6^ found no difference in FI scores between the time-to-event data and frequency data. This finding suggests that the RFI may also not be affected because the results were more sensitive to the number of events in each group rather than to timing of the events. Nevertheless, concerns remain that the RFI might give excessively fragile results in time-to-event data, especially when the events are similar in each group but the timing of events is different. Results of the sensitivity analyses, however, were not statistically significantly different between the included trials, which used time-to-event primary end points vs frequency data. Moreover, the concept of *survival FI,* defined as the number of participants with an event at the mean exposure time of all participants in the study whose addition would result in a loss of statistical significance, has been proposed as an alternative to accounting for events over time.^33^ Methods to calculate FI for logistic regression β coefficients have also been proposed.^33^ These concepts can be extended to RFI in future studies. Third, we analyzed only RCTs with null results that were published in select peer-reviewed journals.

## Conclusions

In this cross-sectional study of the RFI of statistically nonsignificant results of published clinical trials, the median RFI appeared to be low, indicating that a relatively small number of events were required to change to turn the primary end point from nonsignificant to statistically significant. The findings in this study emphasize the nuance required when interpreting trial results that did not meet prespecified significance thresholds.

## Notes

Supplement. **eFigure 1.** Number of Randomized Controlled Trials Within Each Range of Reverse Fragility Index at P-Value Threshold of <0.01 **eFigure 2.** Number of Randomized Controlled Trials Within Each Range of Reverse Fragility Index at P-Value Threshold of <0.005 **eFigure 3.** Scatterplot Illustrating the Correlation Between Sample Size and Reverse Fragility Index at P-Value Threshold of <0.01 **eFigure 4.** Scatterplot Illustrating the Correlation Between Total Events and Reverse Fragility Index at P-Value Threshold of <0.01 **eFigure 5.** Scatterplot Illustrating the Correlation Between Sample Size and Reverse Fragility Index at P-Value Threshold of <0.005 **eFigure 6.** Scatterplot Illustrating the Correlation Between Total Events and Reverse Fragility Index at P-Value Threshold of <0.005 **eTable.** List of Trials Included in the Analysis
