# Supplementary material for: Application of the Reverse Fragility Index to Statistically Nonsignificant Randomized Clinical Trial Results
Source: JAMA Netw Open. 2020 Aug 5;3(8):e2012469. doi: 10.1001/jamanetworkopen.2020.12469 (PMC7407075; doi:10.1001/jamanetworkopen.2020.12469)
Supplement: Supplement. — eFigure 1. Number of Randomized Controlled Trials Within Each Range of Reverse Fragility Index at P-Value Threshold of <0.01 eFigure 2. Number of Randomized Controlled Trials Within Each Range of Reverse Fragility Index at P-Value Threshold of <0.005 eFigure 3. Scatterplot Illustrating the Correlation Between Sample Size and Reverse Fragility Index at P-Value Threshold of <0.01 eFigure 4. Scatterplot Illustrating the Correlation Between Total Events and Reverse Fragility Index at P-Value Threshold of <0.01 eFigure 5. Scatterplot Illustrating the Correlation Between Sample Size and Reverse Fragility Index at P-Value Threshold of <0.005 eFigure 6. Scatterplot Illustrating the Correlation Between Total Events and Reverse Fragility Index at P-Value Threshold of <0.005 eTable. List of Trials Included in the Analysis [file jamanetwopen-3-e2012469-s001.pdf]

Supplementary Online Content

Khan MS, Fonarow GC, Friede T, et al. Application of the reverse fragility index to statistically nonsignificant randomized clinical trial results. *JAMA Netw Open*. 2020;3(8):e2012469. doi:10.1001/jamanetworkopen.2020.12469

eFigure 1. Number of Randomized Controlled Trials Within Each Range of Reverse Fragility Index at P-Value Threshold of <0.01

eFigure 2. Number of Randomized Controlled Trials Within Each Range of Reverse Fragility Index at P-Value Threshold of <0.005

eFigure 3. Scatterplot Illustrating the Correlation Between Sample Size and Reverse Fragility Index at P-Value Threshold of <0.01

eFigure 4. Scatterplot Illustrating the Correlation Between Total Events and Reverse Fragility Index at P-Value Threshold of <0.01

eFigure 5. Scatterplot Illustrating the Correlation Between Sample Size and Reverse Fragility Index at P-Value Threshold of <0.005

eFigure 6. Scatterplot Illustrating the Correlation Between Total Events and Reverse Fragility Index at P-Value Threshold of <0.005

eTable. List of Trials Included in the Analysis

This supplementary material has been provided by the authors to give readers additional information about their work.

eFigure 1. Number of Randomized Controlled Trials Within Each Range of Reverse Fragility Index at P-Value Threshold of <0.01

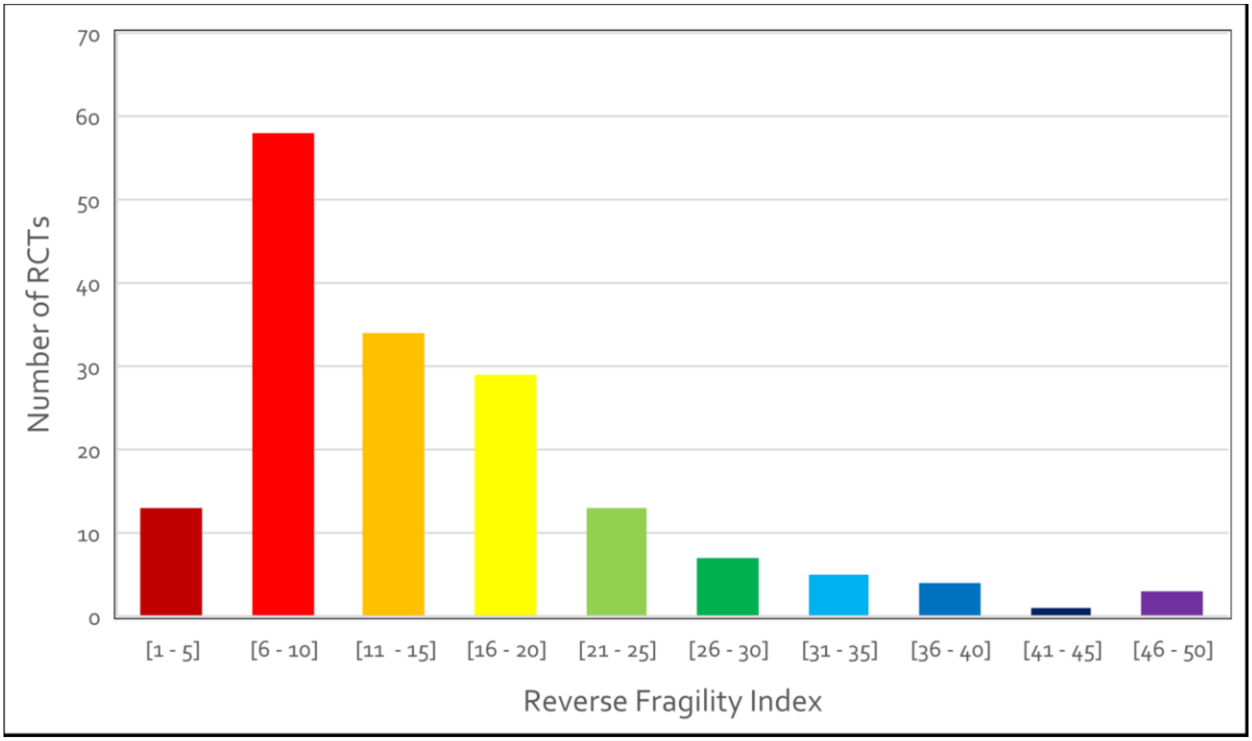

©2020 Khan MS et al. *JAMA Network Open*

eFigure 2. Number of Randomized Controlled Trials Within Each Range of Reverse Fragility Index at P-Value Threshold of <0.005

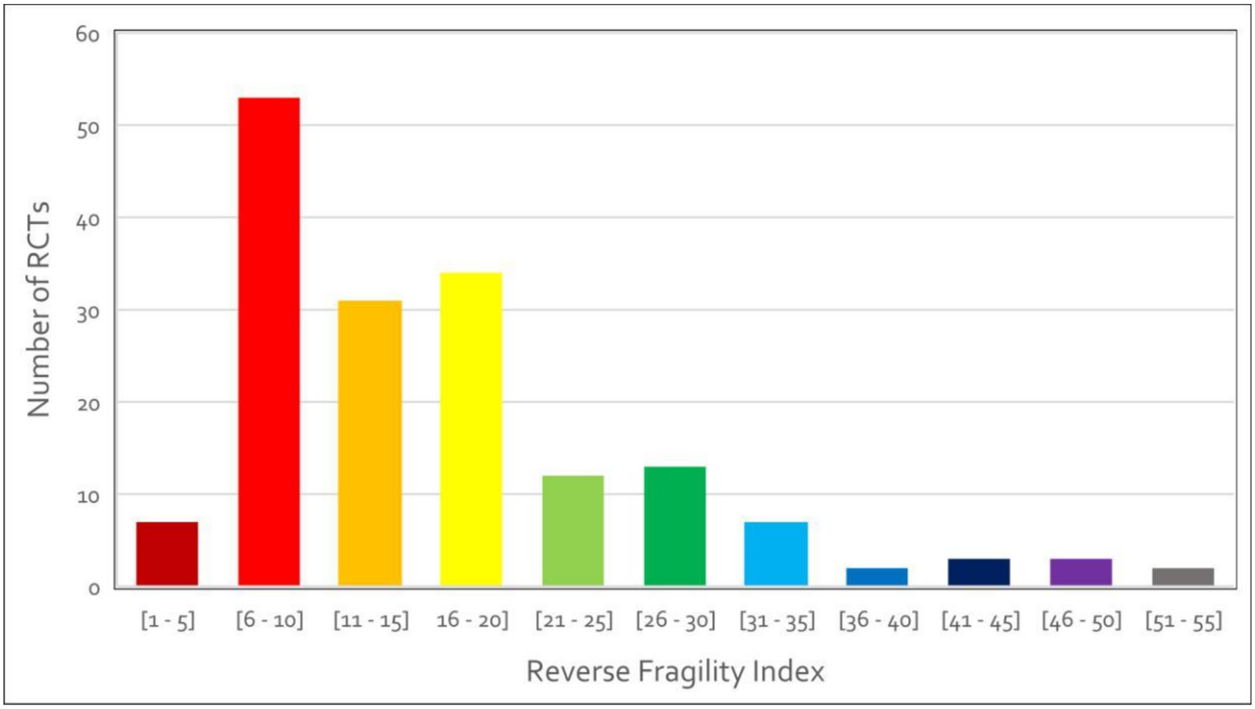

©2020 Khan MS et al. *JAMA Network Open*

eFigure 3. Scatterplot Illustrating the Correlation Between Sample Size and Reverse Fragility Index at P-Value Threshold of <0.01

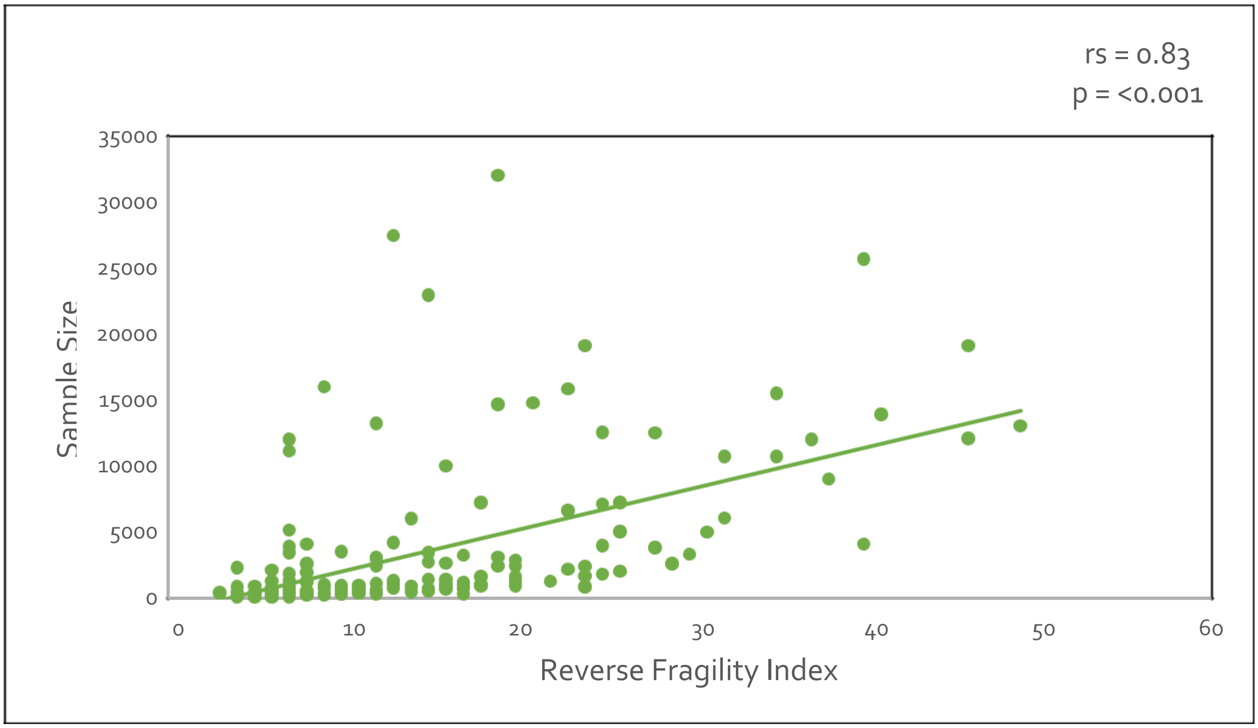

eFigure 4. Scatterplot Illustrating the Correlation Between Total Events and Reverse Fragility Index at P-Value Threshold of <0.01

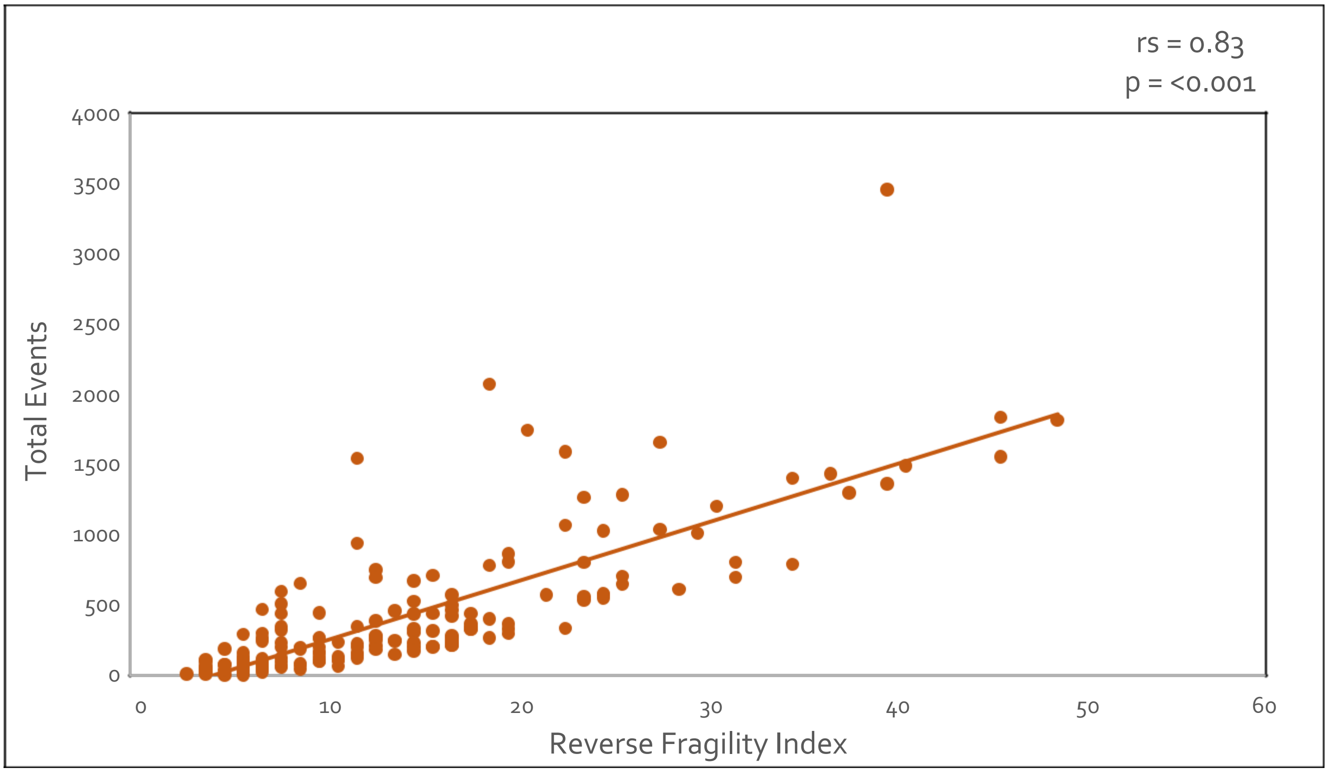

©2020 Khan MS et al. *JAMA Network Open*

eFigure 5. Scatterplot Illustrating the Correlation Between Sample Size and Reverse Fragility Index at P-Value Threshold of <0.005

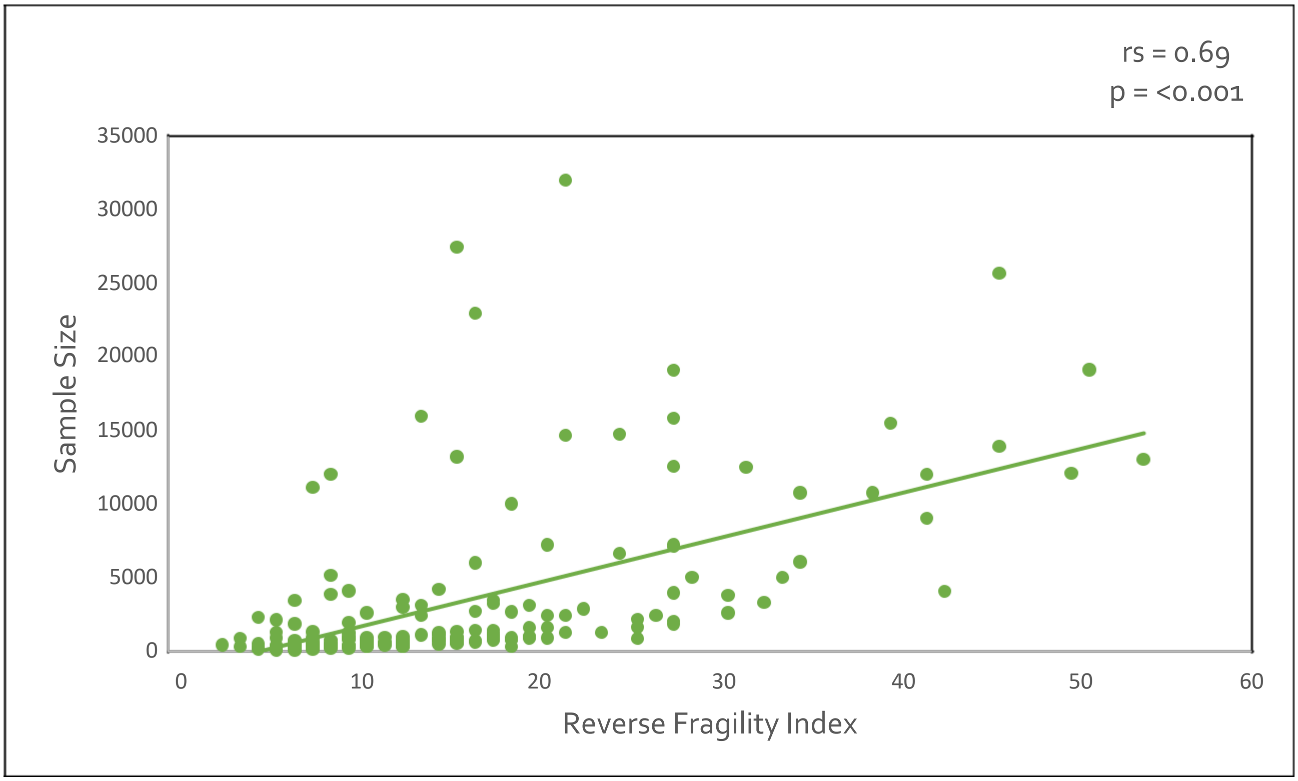

©2020 Khan MS et al. *JAMA Network Open*

eFigure 6. Scatterplot Illustrating the Correlation Between Total Events and Reverse Fragility Index at P-Value Threshold of <0.005

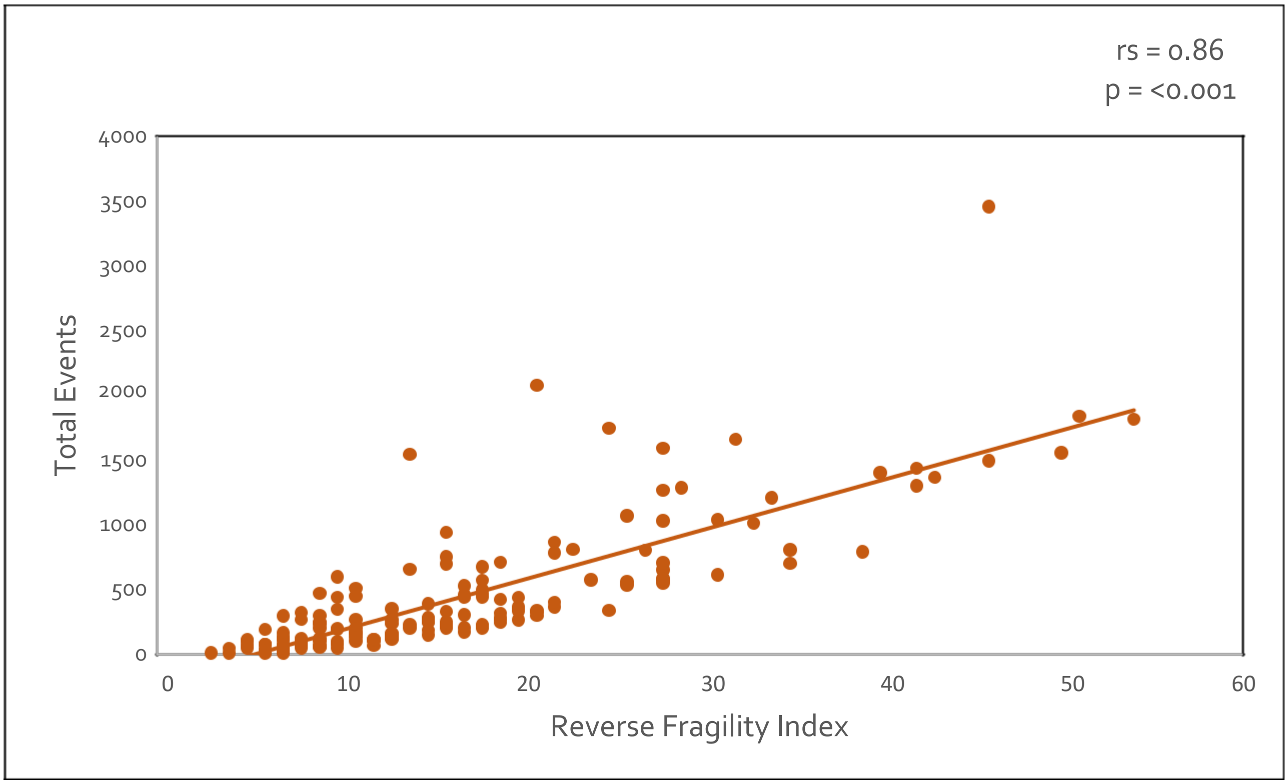

©2020 Khan MS et al. *JAMA Network Open*

eTable. List of Trials Included in the Analysis

Trial Name	PMID
Transfer of Fresh versus Frozen Embryos in Ovulatory Women	29320646
IVF Transfer of Fresh or Frozen Embryos in Women without Polycystic Ovaries	29320655
EPCAT II	29466159
ADRENAL	29347874
iTAP	29514030
EOLIA	29791822
NAVIGATE ESUS	29766772
TRAAP	30134136
CAMELLIA-TIMI	30145941
MARINER	30145946
COMMANDER HF	30146935
IDEAL-ICU	30304656
ASPREE	30221596
ASCEND: A Study of Cardiovascular Events iN Diabetes	30146932
TARGET	30346225
PECARN	30462938
PROGUT	30462939
SUP-ICU	30354950
MITRA-FR	30145927
EUCLID	27959717
THAPCA-IH	28118559
ACCELERATE	28514624
50CHEETAH	28320259
PIVOT	28700844
VALIDATE	28844201
EXSCEL	28910237
DETO2X-AMI	28844200
TRANSFUSE	28952891
ATTRACT	29211671
Australian Placental Transfusion Study	29081267
Intensified Antituberculosis Therapy in Adults with Tuberculous Meningitis	26760084

©2020 Khan MS et al. *JAMA Network Open*

Trial Name	PMID
The Effect of Routine Antibiotic Use in the Outpatient Treatment of Severely Malnourished Children Without Complications	26840134
Adjunctive Dexamethasone in HIV-Associated Cryptococcal Meningitis	26863355
andomized Trial of Labor Induction in Women 35 Years of Age or Older	26962902
A Randomized Trial of a Cervical Pessary to Prevent Preterm Singleton Birth	26981934
EAT	26943128
SOCRATES	27160892
APEX	27232649
Treatment of Fabry's Disease with the Pharmacologic Chaperone Migalastat	27509102
SAVE	27571048
ATACH-2	27276234
DANISH	27571011
NORSTENT	27572953
LOTT	27783918
HYBERNATUS	28002714
ART	27959712
AKIKI	27181456
SPIRE-1 and SPIRE-2	28304242
SECURE-PCI	29525821
PRETTINEO	29715354
Effect of Acupuncture vs Sham Acupuncture on Live Births Among Women Undergoing In Vitro Fertilization: A Randomized Clinical Trial	29800212
SAFE 21	30120476
OMSS	30304425
EUPHRATES	30304428
HIGH	30357270
POLAR-RCT	30357266
J-DAVID	30535217
CAPS	28350929
TOAST	28418482
TOP MRSA	28535235
TTH48	28742911

©2020 Khan MS et al. *JAMA Network Open*

Trial Name	PMID
ASTER	28763550
LICORN	28787507
GUIDE-IT	28829876
ROLARR	29067426
MYRE	29209721
POSTAL	29234808
WIFI	29279933
ABCvitaminD	26813208
Statin AKI Cardiac Surgery	26906014
LATITUDE-TIMI 60	27043082
MOOD-HF	27367876
EMPIRICUS	27706483
HYPRESS	27695824
A Trial of Cranberry Capsules for Urinary Tract Infection Prevention in Nursing Home Residents	27787564
Endoscopic or surgical step-up approach for infected necrotising pancreatitis: a multicentre randomised trial	29108721
NUTRIREA-2	29128300
ARREST	29249276
SCOOP	29254858
TARDIS	29274727
DIVA	29759512
BICAR-ICU	29910040
STAMP	30032978
OSTRICH	30152390
GLOBAL LEADERS	30166073
ARRIVE	30158069
Intra-articular corticosteroids versus intra-articular corticosteroids plus methotrexate in oligoarticular juvenile idiopathic arthritis: a multicentre, prospective, randomised, open-label trial	28162781
FAITH	28262269
ADEPT	28283286
GRECCAR 2	28601342
ATTEND	28666682

©2020 Khan MS et al. *JAMA Network Open*

Trial Name	PMID
TOTAL	26474811
RIVER-PCI	26474810
REGULATE-PCI	26547100
PPROMT trial	26564381
ANRS 12174	26603917
IBIS-II DCIS	26686313
INFORMS	26827074
REMEMBER	27025337
DANAMI 3-DEFER	27053444
TROPHY	27132053
inSIGHT	27132052
REACT-2	27371185
ANTARCTIC	27581531
TITRe2	25760354
PROMISE	25773919
ProMISe study	25776532
TOTAL	25853743
THAPCA-OH	25913022
PermiT trial	25992505
STAN	26267623
SOME	26095467
CIRCUS	26321103
SERVE-HF	26323938
BENEFIT	26323937
RIPHeart-Study	26436208
ERICCA	26436207
PROMISE	26605928
ELIXA	26630143
CORAL	24245566
TOPCAT	24716680
ALBIOS	24635772
SEPSISPAM	24635770
STABILITY	24678955
SAILS	24835849
HPS2-THRIVE	25014686
SIGNIFY	25176136

©2020 Khan MS et al. *JAMA Network Open*

Trial Name	PMID
TRISS	25270275
ARISE	25272316
CALORIES	25271389
PYTHON	25409371
PROTECT III	25493974
PREPIC2	25919526
CATCH	26284719
PIMAL	26348751
SCANDIV	26441181
IVNICTUS	26444879
ASTIC	26670970
LINC Study	24240611
VISTA-16	24247616
CATIS	24240777
AleCardio	24682069
Fluconazole Prophylaxis Study	24794367
START	24794368
ACLAIMS	24846035
VIDA	24838406
OPTIMISE	24842135
JOQUER	25027140
MetaPlus trial	25096691
SOLID-TIMI 52	25173516
A Virtual Ward to Reduce Readmissions After Hospital Discharge	25268437
Levofloxacin for BK virus prophylaxis following kidney transplantation	25399012
FACTOR-64	25402757
FENO HSR	25265449
Japanese Primary Prevention Project (JPPP)	25401325
Neovita trial	25499545
Neovita	25499543
RITP trial	25662413
CONFIDeNT	26293315
EPO-TBI	26452709
SOX Trial	24315521

©2020 Khan MS et al. *JAMA Network Open*

Trial Name	PMID
Infliximab Plus Intravenous Immunoglobulin for the Primary Treatment of Kawasaki Disease	24572997
EAGeR	24702835
PROUD	24718270
PROVHILO trial	24894577
ENIGMA-II	25142708
TIPPS	25066248

©2020 Khan MS et al. *JAMA Network Open*
